# Ketoconazole- and Metyrapone-Induced Reductions on Urinary Steroid Metabolites Alter the Urinary Free Cortisol Immunoassay Reliability in Cushing Syndrome

**DOI:** 10.3389/fendo.2022.833644

**Published:** 2022-02-23

**Authors:** Arturo Vega-Beyhart, Javier Laguna-Moreno, Daniela Díaz-Catalán, Laura Boswell, Mireia Mora, Irene Halperin, Gregori Casals, Felicia A. Hanzu

**Affiliations:** ^1^Group of Endocrine Disorders, Institut d’Investigacions Biomèdiques August Pi i Sunyer, Barcelona, Spain; ^2^Endocrinology and Nutrition Department, Hospital Clinic, Barcelona, Spain; ^3^Biomedical Diagnostics Center, Hospital Clinic, Barcelona, Spain; ^4^Centro de Investigación Biomédica en Red de Diabetes y Enfermedades Metabólicas Asociadas (CIBERDEM), Instituto de Salud Carlos III (ISCIII), Madrid, Spain; ^5^Department of Medicine, Faculty of Medicine and Health Sciences, University of Barcelona, Barcelona, Spain

**Keywords:** cushing syndrome, urinary free cortisol, mass spectrometry, immunoassay, ketoconazole, metyrapone

## Abstract

**Introduction:**

Twenty-four-hour urinary free cortisol (24h-UFC) is the most used test for follow-up decision-making in patients with Cushing syndrome (CS) under medical treatment. However, 24h-UFC determinations by immunoassays (IA) are commonly overestimated because of steroid metabolites’ cross-reaction. It is still uncertain how ketoconazole (KTZ)- and metyrapone (MTP)-induced changes on the urinary steroid metabolites can alter the 24h-UFC*IA determinations’ reliability.

**Methods:**

24h-UFC was analyzed by IA and gas chromatography-mass spectrometry (GC-MS) in 193 samples (81 before treatment, 73 during KTZ, and 39 during MTP) from 34 CS patients. In addition, urinary steroidome was analyzed by GC-MS on each patient before and during treatment.

**Results:**

Before treatment, 24h-UFC*IA determinations were overestimated by a factor of 1.75 (95% CI 1.60–1.94) compared to those by GC-MS. However, during KTZ treatment, 24h-UFC*IA results were similar (0.98:1) to those by GC-MS (95% CI, 0.83–1.20). In patients taking MTP, IA bias only decreased 0.55, resulting in persistence of an overestimation factor of 1.33:1 (95% CI, 1.09–1.76). High method agreement between GC-MS and IA before treatment (*R*^2^ = 0.954) declined in patients under KTZ (*R*^2^ = 0.632) but not in MTP (*R*^2^ = 0.917). Upper limit normal (ULN) reductions in patients taking KTZ were 27% larger when using 24h-UFC*IA compared to 24h-UFC*GC-MS, which resulted in higher false efficacy and misleading biochemical classification of 15% of patients. Urinary excretion changes of 22 urinary steroid metabolites explained 86% of the 24h-UFC*IA interference. Larger urinary excretion reductions of 6β-hydroxy-cortisol, 20α-dihydrocortisol, and 18-hydroxy-cortisol in patients with KTZ elucidated the higher 24h-UFC*IA bias decrement compared to MTP-treated patients.

**Conclusion:**

KTZ and MTP alter the urinary excretion of IA cross-reactive steroid metabolites, thus decreasing the cross-reactive interference of 24h-UFC*IA determinations present before treatment. Consequently, this interference reduction in 24h-UFC*IA leads to loss of method agreement with GC-MS and high risk of overestimating the biochemical impact of KTZ and MTP in controlling CS because of poor reliability of reference ranges and ULN.

## 1 Introduction

Endogenous Cushing syndrome (CS) is characterized by chronic high levels of circulating cortisol caused by either pituitary, adrenal, or ectopic tumors associated with high cardiometabolic morbimortality (1). The first-line treatment of all forms of endogenous CS is surgical resection of the primary tumor. However, tumor resection is often delayed, unsuccessful, or not feasible, resulting in more than 50% of patients with CS requiring medical therapy at some point during the follow-up of the disease (2, 3). Among medical treatments, steroidogenesis inhibitors (SEI) are the drugs most used in CS, reducing cortisol biosynthesis by inhibiting enzymes of the adrenal steroidogenic pathway. Ketoconazole (KTZ) and metyrapone (MTP) have been the most used SEI for decades (4). While KTZ blocks multiple steps of the adrenal steroidogenic pathway through the inhibition of numerous cytochrome p450 enzymes (5), MTP is a more specific inhibitor of the 11β-hydroxylase enzyme, which also blocks 18-hydroxylase to a minor extent (6).

In patients with CS under medical treatment with SEI, clinical follow-up decisions are primarily based on the biochemical control of the disease, which is defined by the normalization of cortisol levels (7). In this regard, twenty-four-hour urinary free cortisol (24h-UFC) is the biomarker most widely accepted to assess the patient’s cortisol levels because it resembles time-integrated tissue exposure to free cortisol over a day; moreover, it is not susceptible to pulsatile secretion and circadian variability as well as changes in cortisol-binding proteins in the serum (8). However, in addition to free cortisol, the urine also contains abundant cortisol metabolites with similar chemical structures. As these metabolites share common antigenic epitopes with cortisol (ring-A cortisol metabolites), cross-reactivity and subsequently overestimated 24h-UFC results are expected when assessed by immunoassay (IA) methods (9). In healthy subjects and patients with active CS, 24h-UFC results obtained by several IA (8, 10–16) have been found to be overestimated by approximately 1.7- to 2.0-fold when compared with those obtained by mass spectrometry (MS), the gold standard method to assess 24h-UFC. Furthermore, serum cortisol determined by IA is also known to be greatly overestimated by MTP treatment due to the accumulation of the circulating steroid 11-deoxycorticosterone (11-DOC) (17–19). Nonetheless, it is unknown if the well-known cross-reactivity interference of 24h-UFC*IA determinations is modified in patients with CS during KTZ or MTP treatment. Moreover, the effects of KTZ and MTP in the urinary excretion of steroid metabolites and their impact on the 24h-UFC*IA bias are also unknown. Therefore, the aim of the present study was to assess how the urinary steroid metabolites changes in patients with CS during medical treatment with KTZ or MTP alter the 24h-UFC determination reliability of IA when compared to GC-MS.

## 2 Methods

### 2.1 Study Design and Participants

The present cohort study included patients with a confirmed diagnosis of *de novo*, persistent, or recurrent endogenous CS (aged >18 years) (20) attended at Hospital Clínic de Barcelona from 2015 to 2019. Consecutive patients fulfilling inclusion criteria were enrolled in the study. Inclusion criteria were at least two adequate (**Supplementary Table 1**) 24-h urine samples before initiation of SEI treatment and at least two adequate 24-h urine samples during the maintenance phase treatment (≥3 months) with therapeutic doses of either KTZ (≥400 mg/day) or MTP (≥500 mg/day). Patients were included in the study and followed up until (1) CS remission was obtained from surgical excision of the tumor, or/and radiotherapy, or bilateral adrenalectomy; (2) death; or (3) December 31, 2020. Patients with CS due to adrenocortical carcinoma were excluded because of possible hypersecretion of multiple adrenal steroid precursors (21). The study design flowchart is shown in **Supplementary Figure 1**. The Institutional Research and Ethics Committees (CEIC) from Hospital Clínic de Barcelona approved the present study (HCB/2019/0179). Written informed consent was obtained from all patients prior to study inclusion. Baseline characteristics of patients are found in **Table 1**.

**Table 1:** Baseline characteristics of patients at the start of treatment.

	Ketoconazole (*n* = 23)	Metyrapone (*n* = 11)	*p*-value
**Age**	46 ± 16	54 ± 15	0.104
**Age diagnosis (years)**	41 ± 16	54 ± 15	**0.025**
**Time symptoms to diagnosis (months)**	7 (4–15)	7 (2–12)	0.714
**Time from diagnosis to metabolite assessment not using SEI**	25 (0–89)	0 (0–24)	0.070
**Cushing syndrome etiology**			
Cushing disease (%)	17 (74)	6 (54)	0.200
Adrenal Cushing (%)	5 (22)	4 (37)	
Ectopic Cushing (%)	1 (4)	1 (09)	
**Sex**			
Female (%)	12 (52)	4 (36)	0.283
Male (%)	11 (48)	7 (64)	
**Number of corrective surgeries**			
None	11 (48)	7 (64)	0.901
1	10 (44)	4 (36)	
2	1 (4)	–	
3	1 (4)	–	
**Use of SEI**			
Neoadjuvant	12 (52)	7 (64)	0.513
Recurrence post-surgery	9 (39)	3 (27)	
Recurrence post-surgery post radiotherapy	2 (9)	1 (09)	
**Basal line tests**			
BMI	25 (23–35)	26 (24–27)	1.0
Glomerular filtrate (CKDPI) ml/min	103 ± 24	106 ± 13	0.982
AST (5–40 U/L)	17 (16–21)	21 (18–38)	0.198
ALT (5–40 U/L)	24 (17–33)	30 (24–38)	0.119
GGT (5–40 U/L)	22 ± 11	30 ± 12	0.714
Serum cortisol (10–25 µg/dl)	19 (16–32)	26 (19–33)	0.216
ACTH (10–60 pg/ml)	30 (16–84)	20 (10–35)	0.283
Late-night salivary cortisol (<1.56 µg/L)	4.5 (2.7–9.5)	7.4 (2.7–19.5)	0.659
24h UFC by IM (20–100 µg/day)	284 (191–795)	790 (293–1455)	0.304
24h UFC by MS (13–60 µg/day)	189 (88–510)	492 (182–1113)	0.150

Normal distributed variables are expressed as mean ± standard deviation. Non-parametrical variables are expressed as median (interquartile range). p-values come from independent t-test or Mann–Whitney U when appropriate. SEI, Steroidogenesis inhibitor; BMI, Body mass index; AST, Aspartate aminotransferase; ALT, Alanine aminotransferase; GGT, Gamma-glutamyl transferase; ACTH, Adrenocorticotropic hormone; 24h UFC, Twenty-four-hour urinary free cortisol.

The bold p-value indicates statistical significance (p < 0.05).

### 2.2 24h-Urinary Samples Selection

24h-urinary samples available during follow-up, including those before SEI, during SEI initiation below therapeutic doses, and while on treatment maintenance phase, were included for each patient. Periodicity of 24h-urinary samples depended on each patients’ clinical need for a follow-up visit (1 month–6 months). 24h urinary samples were collected in a sterile container with no preservative, initiating after the first-morning void, storing it at 2–8°C during the gathering, and concluding with the first-morning void of the following day. An aliquot of 10 ml was preserved at −80°C until analysis. Ninety-five urinary samples from patients before treatment, 81 during KTZ, and 44 during MTP were initially included. We excluded urinary samples that were not adequate (15), assessed by urine volume, creatinine excretion, and glomerular filtration rate (**Supplementary Table 1**). We also excluded urinary samples if the patient was taking any of the following medications within 1 month of the sample collection: corticosteroids; dopamine agonists; synthetic progestins and estrogens; somatostatin analogues; weight loss medications; strong CYP3A4 inducers (e.g., phenytoin and pioglitazone); strong CYP3A4 inhibitors (e.g., clarithromycin, conivaptan, and itraconazole); absorption interferents of KTZ (e.g., histamine H2 receptor antagonists and high doses of proton-pump inhibitors/sucralfate); drugs with systemic exposure increased by KTZ (e.g., HMG-COA reductase inhibitors); and inducers of QTc prolongation. A total of 193 samples were finally included in the analysis: 81 before SEI treatment, 73 during KTZ, and 39 during MTP.

### 2.3 Urinary Free Cortisol and Adrenal Steroid Profile Assessment

UFC*IA measurements were performed following routine methods in our hospital using a chemiluminometric (CM) IA (LIAISON, Diasorin, Italy) after a previous extraction of urine with dichloromethane. UFC*MS measurements were performed by GC–MS as previously described (15). Urinary adrenal steroid profile was measured in one 24-h urine specimen at baseline and one during SEI, following a procedure based on Shackleton et al. (22). The reference standards and the internal standards were obtained from Sigma (Steinheim, Germany), Steraloids Inc (Newport, USA), and NMI (Pymble, Australia). Steroids were extracted from urine with Sep-Pak C18 cartridges (Waters, Milford, MA, USA) and hydrolyzed with sulfatase (Sigma, Steinheim, Germany) and β-glucuronidase/arylsulfatase (Roche Diagnostics, Penzberg, Germany) overnight and re-extracted with Sep-Pak C18 cartridge. The extracts were derivatized with methoxyamine hydrochloride and trimethylsilylimidazole (23, 24). GC-MS analyses were performed on a Shimadzu GC-MS-QP2010 Ultra instrument. Steroids were separated on a Sapiens-5MS+ capillary column (30 m × 0.25 mm internal diameter × 0.25 μm film thickness) from Teknokroma (Barcelona, Spain). The oven temperature conditions were as follows: started at 50°C, maintained at this temperature for 3 min, elevated at 80°C/min to 240°C, increased at 2°C/min until 290°C, and maintained for 4 min at 290°C. The ion source and transfer line temperatures were set to 270°C and 280°C, respectively. Extracts were injected splitless into the chromatographic system and the mass detector was operated in synchronous selected ion monitoring mode.

### 2.4 Statistical Analysis

Linearity, normality, homoscedasticity, and absence of multicollinearity were checked to use the appropriate comparative test. 24h-UFC*IA bias was calculated as 24h-UFC*IA/24h-UFC*MS. (1) 24h-UFC*ULN; (2) control status of the disease (<1.2 ULN = disease control or >1.2 ULN = uncontrolled); (3) 24h-UFC and 24h-UFC*ULN decrease (%) from baseline to maintenance therapy were calculated with 24h-UFC*IA and *MS. Pearson correlation, linear, and non-parametric Passing-Bablok regression analysis were used to compare the performance of IA and MS. Bland–Altman plots were used to test agreement between methods. Independent-samples tests were used to compare baseline characteristics. Pairwise comparisons (Paired *t*-test, Wilcoxon test, McNemar’s test) were employed when testing binomial variables. Linear mixed models with unstructured repeated covariance were used to test for the main effects (maximum likelihood) of SEI (KTZ and MTP) and CS etiology as well as its interactions effects on the 24h-UFC*IA bias. Spearman correlations followed by polynomial regressions with stepwise method were performed to identify those metabolites independently associated with the 24h-UFC*IA bias. Fold changes (FC) were employed to assess the change on each metabolite from baseline to treatment. Post-hoc comparisons were assessed with Bonferroni correction. Pairwise metabolite comparisons employing absolute concentrations during independent tests when appropriated were performed, adjusting each metabolite concentration to each patient’s 24h-UFC*MS concentration to characterize the metabolite effect *per se*, independently of the hypercortisolemia severity. All the comparisons stated as different have statistical significance with *p*-value (two-sided adj. *p* < 0.05). Polynomial models were adjusted for age and sex when needed. Statistical analyses were performed using the R environment 4.1 and SPSS software version 27.

## 3 Results

### 3.1 24h-UFC Performance by IA and GC-MS Before and During Treatment With SEIs

**Figure 1:**
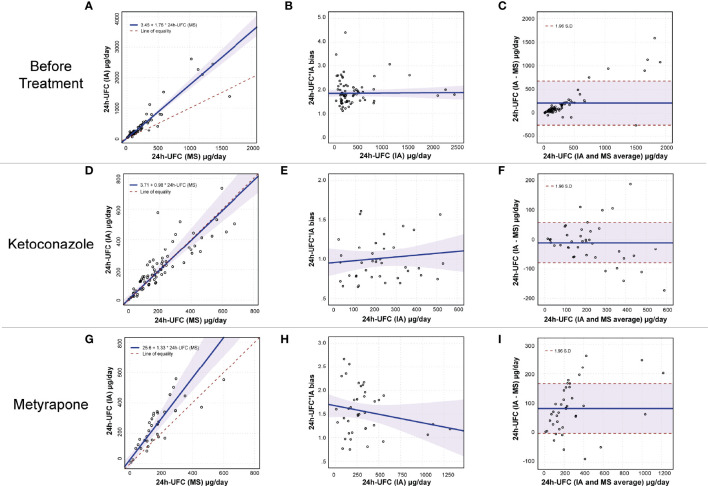
24h-UFC performance method comparison before and during SEI treatment. **(A, D, G)** Passing Bablok regression fit plot (*n* = 81, 73, and 39) between IA and MS before treatment, and during KTZ and MTP, respectively. The 0.95% confidence bounds are calculated with the bootstrap (quantile) method. **(B, E, H)** Regression fit plot for the 24h-UFC*IA bias (*y*-axis) against 24h-UFC*IA concentration, before treatment, during KTZ and MTP, respectively. 95% confidence bounds are calculated to the linear regression method. **(C, F, I)** Bland–Altman plots assessing method agreement on 24h-UFC measurement. 95% confidence bounds are calculated with the bootstrap (quantile) method. 24h-UFC, 24-hour urinary free cortisol; IA, Immunoassay; MS, Mass spectrometry.

Before initiation of the medical treatment for hypercortisolism, 24h-UFC*IA determinations of patients were overestimated by 1.76 (95% CI, 1.60–1.94) when compared to 24h-UFC*GC-MS; however, both methods held a high linear relationship (*r* = 0.977, *R*^2^ = 0.954) (**Figure 1A**). The 24h-UFC*IA bias was constant despite 24h-UFC concentrations (**Figure 1B**) and the agreement between methods was acceptable (**Figure 1C**). When patients were on treatment with KTZ, the 24h-UFC linear relationship between methods sharply decreased (*r* = 0.795, *R*^2^ = 0.632). Moreover, 24h-UFC*IA results were no longer overestimated, giving similar values [0.98 (95% CI, 0.83–1.20)] to those obtained by GC-MS (**Figure 1D**) irrespectively from 24h-UFC concentrations (**Figure 1E**). During KTZ treatment, method agreement between IA and GC-MS was unacceptable as 25% of the 24h-UFC samples lay outside the methods agreement range (**Figure 1F**). These results were independent of CS etiology. Lastly, 24h-UFC*IA and GC-MS determinations from patients under MTP conserved high linear relationship (*r* = 0.958, *R*^2^ = 0.917); however, it was significantly lower in patients with Cushing disease (*R*^2^ = 0.695) than in patients with ectopic CS (*R*^2^ = 0.931) and adrenal CS (*R*^2^ = 0.948). 24h-UFC*IA determinations persisted, overestimated by a factor of 1.33 (95% CI, 1.09–1.76) compared with GC-MS results (**Figure 1G**) without differences between CS etiologies and 24h-UFC*IA concentrations (**Figure 1H**). Thirty-five percent of samples were outside the methods agreement range (**Figure 1I**).

### 3.2 Clinical Implications of the 24h-UFC Determination Differences Between IA and GC-MS

#### 3.2.1 Before Treatment

**Figure 2:**
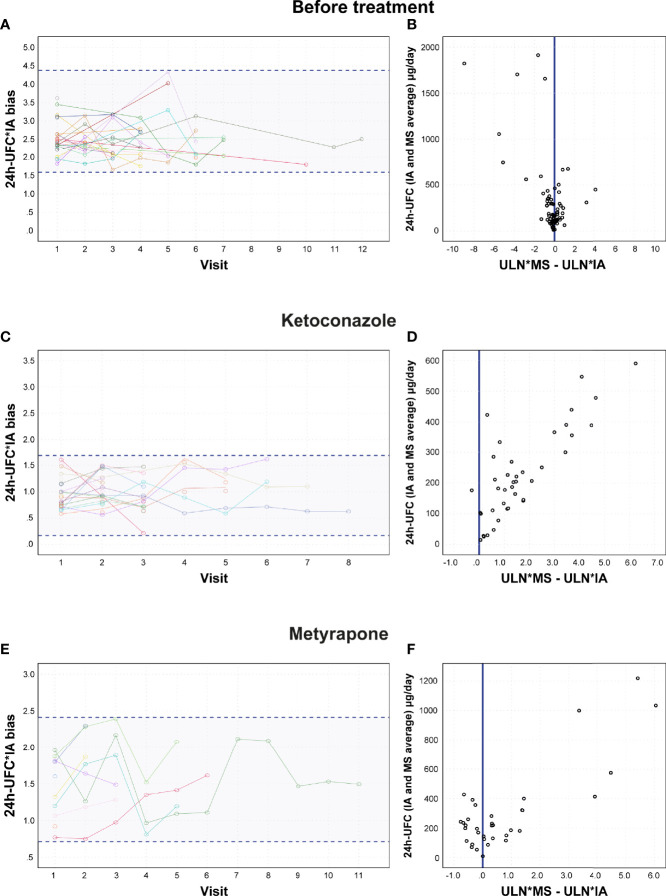
24h-UFC*IA bias variation and ULN differences between methods. **(A, C, E)** Within-subject 24h-UFC*IA bias variation between visits. Each line represents a patient with each node as the 24h-UFC*IA bias of the visit. **(B, D, F)** 24h-UFC ULN difference between methods association plot to 24h-UFC results. 24h-UFC, 24-hour urinary free cortisol; IA, Immunoassay; MS, Mass spectrometry; ULN, Upper limit normal.

Before treatment, mean cohort 24h-UFC concentration assessed by IA was 507.5 μg/day (Min–Max = 137–8343.6), whereas mean cohort concentration by GC-MS was 292.5 (Min–Max = 75.5–4,857.4), resulting in a 24h-UFC*IA bias of 1.84 (1.72–1.97). Within-subject variation of the 24h-UFC*IA bias before treatment was 17% (95% CI, 14–25) (**Figure 2A**). Pairwise comparison of the 24h-UFC*ULN determined by IA vs. GC-MS on each patient revealed no differences between methods (**Table 2**). ULN*MS − ULN*IA difference increased with higher 24h-UFC concentrations (*r* = 0.666, *p* =0.000) although no direction of association was found (**Figure 2B**). In all patients, there was congruency of both methods in the 24h-UFC*ULN biochemical status classification (*p* = 0.508).

**Table 2:** Cortisol response differences between IA and MS.

	Before treatment (*n* = 81)		Ketoconazole^TD^ (*n* = 41)		Metyrapone^TD^ (*n* = 35)	
	IA	MS	IA	MS	IA	MS
24h-UFC*ULN	2.16 (1.37–4.48)	2.08 (1.25–4.22)	2.0 (1.13–3.13)	3.26 (2.04–5.29)	2.69 (1.56–3.7)	2.89 (1.84–4.86)
24h-UFC*ULN (MS–IA)	−0.12 (−0.55–0.25)		1.22 (0.57–2.39)*^┼^		0.18 (−0.36–1.28)*^┼^	
24h-UFC reduction %			63.1 (46.7–80.6)	32.7 (10.6–60.2)	40.1 (26.6–94.5)	38.9 (−38.7–93.3)
24h-UFC reduction % (MS–IA)			−27.9 (−41.4–−12.2)*^┼^		−0.4 (−50.9–8.8)^┼^	
24h-UFC*ULN reduction			2.43 (1.09–6.29)	1.13 (0.4–3.52)	3.18 (1.08–10.2)	4.05 (−0.56–10.15)
24h-UFC*ULN reduction (MS–IA)			−1.18 (−2.92–−0.56)*		−1.12 (−3.87–0.98)	
Creatinine urinary excretion (mg/24 h)	1,154.0 (947–1,308)		1,143.5 (873.5–1,294.5)		1,158 (1,030–1,375)	

Cohort median (interquartile range) values are displayed. *p-value < 0.05 from two-related sample Wilcoxon test (Baseline–Treatment). ^┼^p-value < 0.05 from independent post-hoc intergroup comparison analysis (Bonferroni) between Ketoconazole vs. Metyrapone group. 24h-UFC, Twenty-four-hour urinary free cortisol; MS, Mass spectrometry; IA, Immunoassay; TD (superscript), Therapeutic dose ≥ 400 mg/day.

#### 3.2.2 During KTZ Treatment

**Figure 3:**
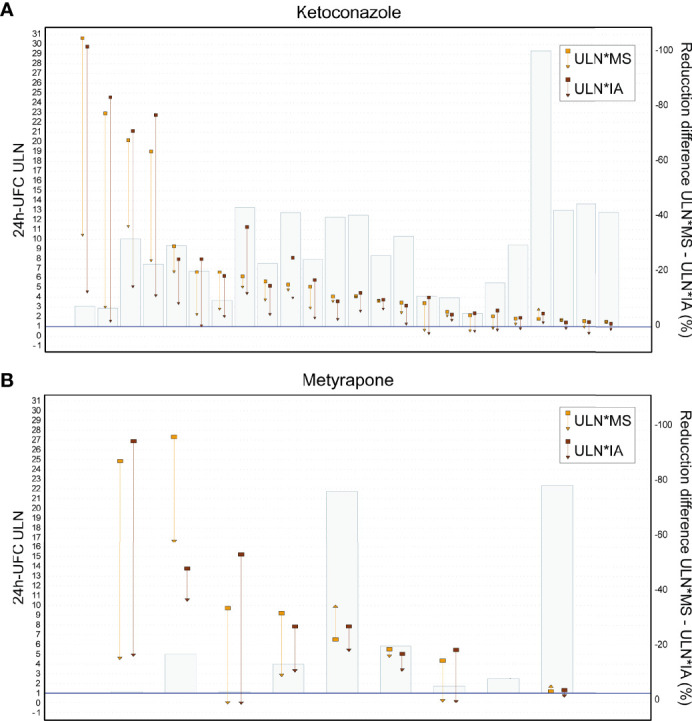
24h-UFC ULN reduction differences between methods. **(A)** Patients with Ketoconazole. **(B)** Patients with Metyrapone. Each patient’s 24h-UFC ULN change is displayed by a pair of vertical arrows (IA and MS). Squares are baseline 24h-UFC ULN to triangles on end of maintenance phase. Bars are the difference % of the 24h-UFC ULN*MS–24hUFC ULN*IA. 24h-UFC, 24-hour urinary free cortisol; IA, Immunoassay; MS, Mass spectrometry; ULN, Upper limit normal.

24h-UFC*IA results were equally overestimated in patients taking low doses of KTZ (100–<400 mg/day) as before treatment. However, in patients taking therapeutic doses (KTZ >400 mg/day) the 24h-UFC*IA bias decreased 0.94 (95% CI [0.69–1.24]) in a dose-dependent way (*p* = 0.000) (**Supplementary Figure 2**). Thus, 24h-UFC*determinations were similar to those by GC-MS with a minimum residual bias of 1.01 ± 0.30 (*p* = 0.000). KTZ explained 40% of the variance of the 24h-UFC*IA cross-reactivity interference (*p* = 0.000) and no interaction effect between KTZ and CS etiology was found (**Table 3**). The decrease in the 24h-UFC*IA bias was independent of treatment duration (*p* = 0.890). Within-subject variability of the 24h-UFC*IA cross-reactivity interference during the maintenance phase with KTZ was 17.1% (95% CI, 11–23) (**Figure 2C**). Comparison of 24h-UFC*ULN between methods in each patient revealed that ULN*IA was lower (*p* = 0.000) than ULN*MS (**Table 2**), which caused a discordant classification of the biochemical control status in 6 patients (15.0%, *p* = 0.035). ULN*MS − ULN*IA difference in patients taking KTZ (1.22 [0.57–2.39]) was larger than the one found in patients before treatment (*p* = 0.000) and kept incrementing as 24h-UFC concentrations increased (*r* = 0.791, *p* = 0.000) (**Figure 2D**). Improvements in hypercortisolism evaluated as the 24h-UFC reduction were falsely higher by 27.9% (12.2%–41.4%) when assessed by IA vs. GC-MS (**Table 2** and **Figure 3A**). These differences resulted in 10 patients (29%) having a >50% ULN reduction from baseline only when calculated with IA (*p* = 0.002). In patients with KTZ treatment, ULN reduction differences between methods were independent from baseline 24h-UFC concentrations (*p* = 0.812). Previous parameters during KTZ did not differ among CS etiologies.

**Table 3:** 24h-UFC*IA bias changes from baseline to maintenance phase of KTZ or MTP treatment.

Fixed effects	Estimate (95% CI)	Sth. Error	*t*	Sig.
**Ketoconazole**				
KTZ	−0.969 (−1.248 – −0.691)	0.097	−8.632	0.000
KTZ * Cushing disease	0.02 (−0.539 – 0.58)	0.282	0.072	0.943
KTZ * adrenal CS	−0.07 (−0.829 – 0.688)	0.383	−0.184	0.855
KTZ*ectopic CS	−0.243 (−0.517 – 0.032)	0.139	−1.747	0.083
**Metyrapone**				
MTP	−0.555 (−1.211 – 0.141)	0.331	−1.678	0.096
MTP * Cushing disease	−0.716 (−1.23 – −0.201)	0.26	−2.756	0.007
MTP * adrenal CS	−0.946 (−1.432 – −0.459)	0.246	−3.852	0.000
MTP* ectopic CS	−0.230 (−0.575 – 0.015)	0.174	−1.322	0.189

Estimates were calculated based on the maximum likelihood change from baseline 24h-UFC*IA bias. All results come from a generalized linear mixed model (LMM) with repeated measurements adjusted for age. Main effects were calculated for metyrapone and ketoconazole. Interaction effects between CS etiology and each medical treatment are also shown. 95% CI for the mean change. t and Sig. values from LMM. Std. Error, Standard error for estimate; Sig, Significance; KTZ, Ketoconazole; MTP, Metyrapone; CS, Cushing syndrome.

#### 3.2.3 During MTP Treatment

In patients taking MTP, the 24h-UFC*IA bias did not decrease in patients with ectopic CS and differently decreased in patients with Cushing disease than in those with adrenal CS (**Table 3**). 24h-UFC*IA determinations during MTP follow-up in patients with Cushing disease were overestimated by a factor of 1.64 (1.20–1.81), while in patients with adrenal CS, by 1.35 (1.09–1.61), both differing from those found on patients with ectopic CS, which were overestimated by 2.35 (1.97–2.61) (*p* < 0.01). Neither MTP dose (250 mg–3 g) nor treatment duration was associated with the 24h-UFC*IA bias decrease rate. Within-subject variability of the 24h-UFC*IA cross-reactivity interference during MTP treatment was 19% (95% CI [10–27]) (**Figure 2E**). Post-hoc analysis showed that MTP lowered the 24h-UFC*IA bias 0.55 less (95% CI [0.30–0.80]) than KTZ > 400 mg/day (*p* = 0.000). 24h-UFC*ULN comparison between methods in each patient revealed that ULN*MS was 0.93 higher (95% CI, 0.32–1.54]) than ULN*IA. ULN*MS − ULN*IA differences were larger than when not treated (*p* = 0.016) but not distinct than in patients with KTZ (*p* = 0.364). ULN*MS − ULN*IA differences were also larger with higher 24h-UFC concentrations (*r* = 0.626, *p* = 0.000) (**Figure 2F**). When comparing MTP efficacy between methods, ULN baseline reductions were >50% different between IA and GC-MS in some patients, while in others, similar reductions were found (**Figure 3B**). Because of the large variance between subjects treated with MTP, no statistical difference was found when performing pairwise comparison of each patient ULN and 24h-UFC decrement assessed by IA vs. GC-MS (**Table 2**).

### 3.3 SEI Treatment Induced-Changes on Urinary Adrenal Steroid Profile and Its Association With 24h-UFC Determination Differences Between Methods

**Figure 4:**
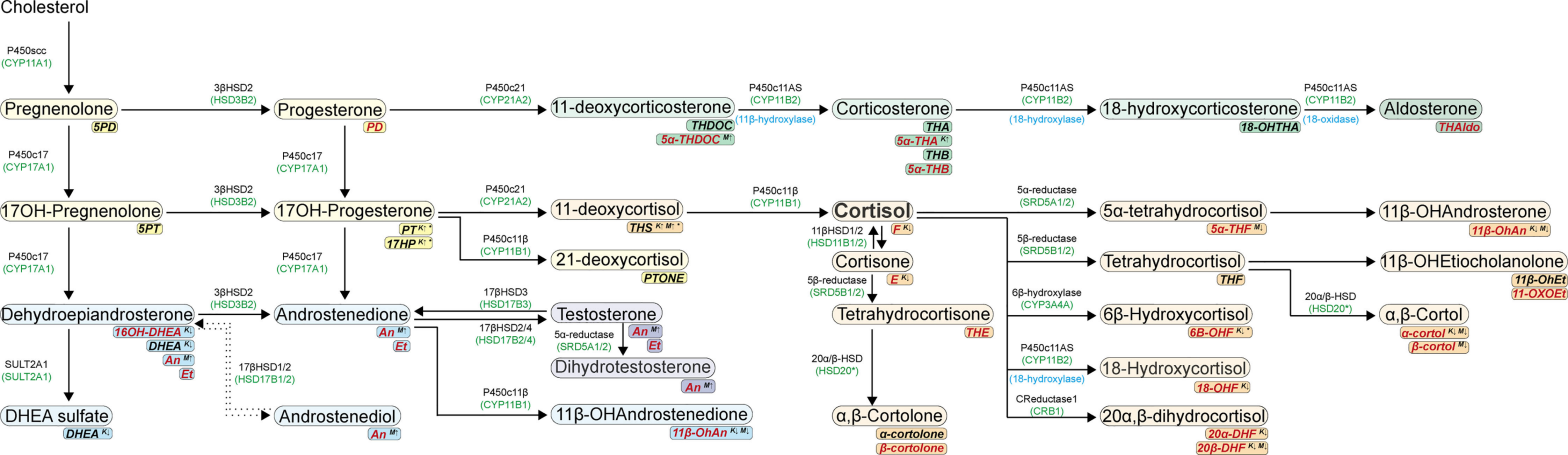
Urinary adrenal steroid portray changes by ketoconazole and metyrapone. Steroids are colored according to their bioactivity: Glucocorticoid precursors (yellow), mineralocorticoid precursors (green), active mineralocorticoid (dark green), active glucocorticoid (orange), androgen precursors (light blue), and active androgens (dark blue). Corresponding urinary metabolites are shown in italic font below each steroid. Black font urinary metabolites are those not associated to the 24h-UFC*IA bias while those in red are. Superscript K (ketoconazole) and M (metyrapone) identify those urinary metabolites with significant concentration change during treatment. Arrow following K or M superscript indicates change direction during treatment.

Regardless of the CS etiology or medical treatment status, the urinary abundance of 22 metabolites explains the 86% of the 24h-UFC*IA cross-reactivity interference fluctuation (*p* = 0.000) (**Table 4** and **Figure 4**). 6β-Hydroxy-cortisol (6β-OH-cortisol) was the metabolite determining for most of the 24h-UFC*IA bias variability (*R*^2 =^ 48.3%, *p* = 0.000) followed by 20α-dihydrocortisol (20α-DHF) (*R*^2 =^ 24.4%, *p* = 0.000) (**Supplementary Figure 3**). The FC of 14 metabolites was associated with the decrease of the 24h-UFC*IA bias found in patients during treatment with SEI (**Table 4**). As 24h-UFC*IA determinations were less overestimated in patients taking KTZ than in those with MTP, we then searched for FC differences in the cross-reactive metabolites among treated groups. Baseline metabolites’ concentration and during treatment with KTZ or MTP as well as mean FC are displayed in **Table 5**. Among GC cross-reactive metabolites, 18-OH-Cortisol and 20α-DHF concentrations decreased more than 50% in patients with KTZ, while no significant reductions were observed in those with MTP. Conjugated cortisol and corticosterone also declined in KTZ (FC = 0.72 and 0.63 respectively, *p* < 0.01) but not in patients under MTP. Moreover, 6β-OH-cortisol urinary excretion was reduced by 80% in patients under KTZ (*p* < 0.001) while no statistical change was found in patients with MTP. Higher KTZ doses were linearly associated with larger reductions of the GC cross-reactive metabolites 6β-OH-cortisol, cortisone, conjugated cortisol, 11-Oxo-etiocholanolone, and 11β-Hydroxy-androsterone; however, no association with MTP dose was found (**Supplementary Figure 4**). On the other hand, β-cortol and 5αTHF were the only GC metabolites that reduced in MTP (FC = 0.71 and 0.64) and not in KTZ (FC = 1.2 and 0.80). Moreover, a 90%–130% increase in concentration of GC precursors 17-OH-pregnanolone (17HP), pregnanediol (PD), and pregnanetriol (PT) was observed in patients after taking KTZ, while no difference was seen in patients after MTP (**Table 5**). However, mean increase from baseline concentrations in tetrahydro-11-deoxycortisol (THS) was over 400% in patients with MTP, while only a 95% increase was found in those under KTZ (**Table 5**). 5α-tetra-hydro-deoxycorticosterone was the only mineralocorticoid that increased as a result in patients with MTP with a mean concentration increment >300%. However, 5α-tetra-11-dehydrocorticosterone had a 50% augment in those with KTZ (**Table 5**). Portrayal of the urinary adrenal steroid changes by KTZ and MTP is displayed in **Figure 4**.

**Table 4:** Adrenal steroid metabolites determining the 24h-UFC*IA bias.

	Linear regression model					FC correlations	
	*β*	95% CI		Sth. Error	*p*	Correlation coefficient	*p*
		LL	UL				
**Androgens**							
Androsterone	−0.109	−0.144	−0.075	0.017	0.000	0.427 *^8^*	0.021
Etiocholanolone	0.092	0.059	0.124	0.016	0.000	0.357 *^12^*	0.042
**Androgen precursor**							
Pregnenediol	−0.693	−1.223	−0.163	0.259	0.012	0.096	0.615
16-Hydroxy-Dehydroepiandrosterone	−0.071	−0.117	−0.026	0.022	0.003	0.353 *^13^*	0.040
**Glucocorticoid precursor**							
Pregnanediol	0.116	0.065	0.166	0.025	0.000	0.305 *^14^*	0.046
**Glucocorticoids**							
11-Oxo-etiocholanolone	−0.039	−0.076	−0.002	0.018	0.041	0.450 *^6^*	0.013
11β-Hydroxy-androsterone	0.047	−0.008	0.101	0.027	0.092	0.288	0.122
Tetrahydrocortisone	0.008	0.002	0.014	0.003	0.012	0.193	0.306
Cortisone	−0.239	−0.359	−0.119	0.059	0.000	0.438 *^7^*	0.016
5α-Tetrahydrocortisol	0.037	0.021	0.052	0.008	0.000	0.381 *^10^*	0.042
β-Cortol	−0.035	−0.053	−0.017	0.009	0.000	0.231	0.220
β-cortolone	−0.054	−0.080	−0.029	0.013	0.000	0.567 *^4^*	0.002
α-cortol	0.041	0.015	0.067	0.013	0.003	0.366 *^11^*	0.047
6β-Hydroxy-cortisol	0.144	0.005	0.283	0.068	0.043	0.681*^1^*	0.000
18-Hydroxy-cortisol	−0.089	−0.132	−0.046	0.021	0.000	0.471 *^5^*	0.011
20α-dihydrocortisol	0.013	0.006	0.019	0.003	0.001	0.594 *^2^*	0.001
20β-dihydrocortisol	0.083	0.034	0.131	0.024	0.001	0.570 *^3^*	0.002
**Mineralocorticoids**							
5α-Tetrahydrodeoxycorticosterone	0.349	0.196	0.502	0.075	0.000	0.395 *^9^*	0.034
Tetrahydrocorticosterone	−0.004	−0.009	0.000	0.002	0.076	0.176	0.362
5α-Tetra-11-dehydrocorticosterone	−0.487	−0.787	−0.186	0.147	0.002	0.144	0.456
5α-Tetrahydrocorticosterone	−0.129	−0.256	−0.003	0.062	0.045	0.080	0.682
Tetrahydroaldosterone	−0.104	−0.173	−0.034	0.034	0.005	0.046	0.313

Multivariate linear regression model was adjusted for age and sex (F = 8.138, p = 0.000, R^2^ = 0.861). Dependent variable of the model is the 24h-UFC*IA bias. β coefficient represents the degree of change in the metabolite concentration for every 1-unit of change in 24h-UFC*IA bias. 95% CI, confidence intervals for coefficient β. LL, Lower level. UL, Upper level. p-value and Std. Error for calculated β in the model.

Superscript numbers on the correlation coefficient of each metabolite indicate order of significance of association with 24h-UFC*Bias

**Table 5:** Urinary metabolites abundance changes during treatment with SEIs.

	Before treatment	Ketoconazole		Metyrapone	
	Concentration (µg/day)	Concentration (µg/day)	Fold Change	Concentration (µg/day)	Fold Change
**Androgen precursor**					
16–Hydroxy–Dehydroepiandrosterone	55.4 (23–90.6)	26.5 (14.9–64.5)	0.56*	47.9 (28.8–93.3)	0.97
5–Pregnenetriol	314.6 (110.1–738.3)	512.7 (177.8–1191.2)	1.42	214 (77.7–750.1)	0.92
Dehydroepiandrosterone	58.2 (18–571.1)	30.4 (16–65)	0.62**	129.3 (32.8–1033.4)	0.99
Pregnenediol (5–pregnene–3b20a–diol)	58.1 (30.1–94.7)	63 (39.5–92.6)	1.04	47.7 (38–137.4)	0.97
**Androgens**					
Androsterone	1048.2 (612–1440.5)	986.4 (771.2–1621.5)	0.99	1456.7 (600–3191.7)	1.51*
Etiocholanolone	1290.1 (845.6–2163.7)	1810.3 (1269.4–2437.4)	1.02	740.4 (369.1–3265.3)	0.99
**Glucocorticoid precursor**					
17–OH–pregnanolone	374.5 (194.6–583.8)	875.6 (318.5–1469.3)	1.94**	361.6 (173.6–823.9)	1.06
Pregnanediol	327.1 (158.7–438.6)	627.5 (285.3–1010.6)	2.18***^┼^	427 (90.4–769.9)	1.12
Pregnanetriol (PT)	507.3 (303.9–868.7)	1352.8 (645.4–2350.2)	2.35***^┼^	430.8 (318.8–1396.4)	1.11
Pregnanetriolone	91.2 (46.9–184.7)	71.6 (41.1–169.9)	1.12	69.8 (33.1–349.9)	1.37
Tetrahydro–11–deoxycortisol	3187.7 (1013.1–7197)	7200.4 (2388–12352.8)	1.95*^┼^	28993.3 (4405–100941)	4.57**
**Glucocorticoids**					
11–Oxo–etiocholanolone	784 (252.8–922.7)	856.5 (297.8–1144.6)	0.99	510.8 (116–1683.7)	0.87
11β–Hydroxy–androsterone	1046.4 (527.3–1853.8)	521.4 (362.3–864)	0.53**	496.3 (397.8–1113)	0.59*
18–Hydroxy–cortisol	602.2 (176.9–1487.8)	242.3 (134.2–410.1)	0.38**	203.7 (94.2–1054.2)	0.29
1β–Hydroxy–etiocholanolone	572.5 (240.2–916.3)	547.5 (397.7–1061.8)	0.92	446.2 (52.8–1324.5)	0.76
20α–dihydrocortisol	15993.8 (4917.5–32167.8)	4974.6 (3896.5–8183.1)	0.48***	6716 (2066.2–23829.3)	0.8
20β–dihydrocortisol	1976.1 (930.1–3565.9)	1053.4 (840.9–1721.8)	0.73**	1085.6 (513.2–2327.4)	0.59*
5α–Tetrahydrocortisol	3226.4 (1789.9–9248.7)	2626 (1215.4–4172.1)	0.8	4061.7 (1487.6–5269.6)	0.64*
6β–Hydroxy–cortisol	283.5 (125.6–740.3)	62.1 (31.3–109.8)	0.21***^┼^	121.2 (42.9–297.1)	0.42
Cortisol	534.2 (301.2–1031.1)	405.5 (272.1–584.2)	0.72**	319.7 (243.7–867.1)	0.79
Cortisone	422.2 (222.8–974.4)	383.5 (197.6–476.7)	0.63**	389.2 (156.6–897.8)	0.81
Tetrahydrocortisol	8730.4 (4211.9–11508.9)	7900.8 (4106.4–11467.7)	0.8	6847.9 (2063–16499.7)	0.81
Tetrahydrocortisone	10023.2 (6042–18295.6)	12089.5 (6314.6–14920.3)	0.98	11672.8 (5140.2–17625.2)	0.81
α–cortol	4504.4 (2746.2–6551.9)	3326.8 (2555.5–5809.9)	0.72*	3146 (2137.7–10115.1)	0.81*
α–Cortolone	4048.1 (2805.5–5348.8)	3798.3 (2912.5–5032.4)	0.97	3707.6 (2521.8–7992.3)	0.96
β–Cortol	2051.7 (1094.3–3957.6)	2431.6 (1438.5–4362.9)	1.22	1588.9 (832.9–3668.3)	0.71*
β–cortolone	4015.6 (2786.1–5303.6)	3767.4 (2885.5–5014.8)	0.97	3675.6 (2499.6–7929.5)	0.95
**Mineralocorticoids**					
5α–Tetra–11–dehydrocorticosterone	55.8 (31.3–68.5)	88.5 (65.5–131)	1.5**	66.1 (46–114.8)	1.38
5α–Tetrahydrocorticosterone	601.3 (215.1–1198.7)	700.4 (399.1–1034.4)	0.92	498.1 (326.5–1224.3)	0.85
5α–Tetrahydrodeoxycorticosterone	76.5 (46.4–113.6)	106.2 (48.7–187.9)	1.38	133.6 (84.6–517.4)	3.52*
Tetrahydro–11–dehydrocorticosterone	167.5 (67–282.8)	153.2 (111.9–227.4)	1.34	121.8 (59–700.8)	0.89
Tetrahydroaldosterone	19.5 (8.3–56.9)	22.4 (9.3–53)	0.68	18.8 (7–45.1)	1.11
Tetrahydrocorticosterone	2500.7 (885.1–5867.4)	3894.8 (2129.9–14887.3)	1.47	2389.5 (649.4–4704.6)	0.72
Tetrahydrodeoxycorticosterone	85.1 (53.2–147.9)	120.8 (74.9–207.6)	1.55	122.9 (92.1–218.4)	1.4

Before treatment concentrations are displayed as a unique group because no differences were found in any metabolite between other groups according to its treatment. *p < 0.05; **p < 0.01; ***p < 0.001 (Hochberg adjustment applied to p-values to control type 1 error at 0∙05) from Wilcoxon or paired t-test were applied accordingly to distribution of the data. ^┼^p < 0.05 from Mann–Whitney U, comparing FC from patients under ketoconazole vs. metyrapone.

## 4 Discussion

In the present study, we described how the unique changes induced by KTZ or MTP on the urinary steroid profile differently altered the performance of the 24h-UFC*IA in a cohort of patients with CS. We identified that IA bias overestimation of 75% found in patients before treatment decreased to almost 0% in patients taking KTZ, while it decreased to 33% in those with MTP therapy. Reductions of ULN were magnified by 27% when using IA compared to GC-MS. These results led to the false categorization of 15% of patients taking KTZ as biochemically controlled when using ULN*IA. Furthermore, 24h-UFC*ULN differences between methods were more significant in patients taking KTZ than MTP. Finally, we demonstrated that 86% of the extent of the 24h-UFC*IA cross-reactivity interference was explained by the abundancy variation of 22 steroid metabolites and that the different degrees of 24h-UFC*IA bias found in patients with KTZ vs. MTP were caused by distinct reductions of urinary metabolites like 6β-OH-cortisol.

Though our results are only based on the IA cortisol LIAISON Diasorin kit, studies employing other IA [Siemens ADVIA Centaur XP (17); Gamma Coat CA 1529, kit A and Spectria cortisol RIA, kit B (18), Roche CM cortisol IA (19), Access Cortisol IA (12)] have also found cross-reactivity interference of steroid metabolites in the cortisol determination. A recent letter by Perrin et al. (10) mentions that a CM microparticle IA using reagent ARCHITECT Cortisol (Abbott Diagnostics) is reliable for 24h-UFC follow-up in CS patients on SEI if the same technique is used before and during treatment. This statement should be taken carefully, as Abbot IA overestimated 24h-UFC by a factor of almost 2:1 in healthy controls and CS patients without any SEI treatment. In contrast, in patients taking KTZ or MTP, IA cross-reactivity interference was entirely abolished, giving similar 24h-UFC values to those obtained by LC-MS. These results suggest the same pattern of bias decrement in the IA during treatment with SEI as observed in our IA, which could lead to a similar false magnification of KTZ or MTP efficacy as observed in our patient. Establishing specific ranges of normality for patients taking SEI would not solve IA poor performance as we observed a non-linear decrease in the cross-reactivity interference that depended on KTZ dose. This would make it necessary to establish several limits of normal values. The wide intraindividual variation in the IA bias found even before treatment could be associated with day-to-day variability in the steroid secretion, as it occurs with cortisol (25, 26). In fact, like Wood et al. (16) using Coat-A-Count Cortisol and ADVIA Centaur IA, we found that the 24h-UFC*IA bias was not associated with the 24h-UFC concentrations but with the change in the urinary abundance of several metabolites. Nonetheless, Bianchi et al. (12) found higher interference with increasing 24h-UFC concentrations using the Access Cortisol IA, which corroborate performance variability among IA.

24h-UFC has been the most used biomarker to assess the normalization of cortisol secretion and, therefore, to study the efficacy of SEI (6, 27–32). Clinical trials and studies evaluating MTP efficacy (6, 29, 30) have employed MS to measure 24h-UFC. However, comparisons with studies that determined MTP efficacy by 24h-UFC*IA (31–33) and with centers following patients with IA would lead to false conclusions, as we found that ULN*MS tend to be 0.96 higher than ULN*IA, increasing with higher 24h-UFC concentrations. Contrary to date, no study (31, 34–41) has assessed KTZ efficacy employing MS in the 24h-UFC determinations. In our patients with KTZ, 24h-UFC reductions by IA were 27.9% larger than by GC-MS, resulting in 29% of patients having >50% reduction of the ULN and in 15% being classified as biochemically controlled, different to GC-MS results. Therefore, parameters resulting from 24h-UFC determinations should be carefully considered because even when ULN are normalized to each kit reference range of normality, the change in the bias that KTZ causes in each IA could lead to different results. However, we previously published that in terms of CS diagnosis, both methods present a very similar diagnostic value (15). However, a detailed analysis of the receiver operating characteristic curves pointed out that, at the same sensitivity, low levels of UFC are more specific when measured by IA; on the contrary, high levels of UFC, at the same specificity, are less sensitive when measured by IA. This suggests that 24h-UFC*IA might be more useful for CS screening and that 24h-UFC* might be more valuable for excluding CS.

Studies assessing serum cortisol cross-reactions (17, 19, 42) have led to the recommendation of using IA with high antibody specificity (10) without interference for adrenal precursors known to accumulate during KTZ (17-OHP) or MTP (11-DOC and 21-DOC) treatment (43, 44). However, we found that, in urine, the abundance of 22 adrenal metabolites determined 86% of the degree of IA interference, pointing out that cortisol IA determinations are interfered by several metabolites (45) rather than only by those stated in current practice guidelines for the use of SEI (3–5, 46, 47). In our group of patients, 6β-OH-cortisol, 20α-DHF, 20β-DHF, β-cortolone, and 18-OH-cortisol were the metabolites with more IA interference. Inhibition of 6β-hydroxylase by KTZ (48) produced an 80% decrease of 6β-OH-cortisol, whereas changes by MTP were not found. 20α-DHF and 18-OH-cortisol were also significantly reduced only in patients with KTZ in accordance with the inhibitory activity against 11β-HSD1 and P450c11AS (49). These unique changes explained most of the reduction difference in 24h-UFC*IA bias between KTZ and MTP, though other changes of precursor’s metabolites were also associated. KTZ induced changes in the urinary excretion of 44% of the adrenal steroids, while it was 23% for MTP. As expected, accumulation over 100% of 17-OH-pregnanolone 17HP, PD, and PT was found in patients with KTZ (42, 44). 11-DOC metabolite THS was also augmented in patients with KTZ by 100%. Nonetheless, patients with MTP had urinary increments of over 300% of THS and 11-DOC metabolite 5α-THDOC, resembling the potent inhibition on the 11β-hydroxylase enzyme (7). MTP-treated patients also had an accumulation of androsterone with an excretion increase of 50%, supporting the documented accumulation of mineralocorticoid and androgens by MTP (28).

Limitations of the study arose from the differences in pre-analytical treatments, transition modes, and calibration of MS that introduce sources of variability in the cortisol measurement between centers. However, the use of standardized methodology across laboratories should tackle these inconveniences (9). On the other hand, the consistent results from a large number of samples of different medical treatment scenarios of patients with CS under KTZ or MTP are strengthened and point out the loss of reliability of IA in patients under SEI treatment when compared to the gold standard MS.

## 5 Conclusion

Different degrees of cross-reaction interference on 24h-UFC*IA determinations before treatment and during KTZ or MTP make IA less suitable for cortisol evaluation. The loss of bias brings overestimated reductions of 24h-UFC, magnifying the efficacy of the medical SEI. We encourage authors to be aware of the 24h-UFC method when comparing SEI efficacy results, as biased conclusions could occur when evaluating efficacy with IA. Moreover, clinicians should take into consideration that 24h-UFC*IA determinations are no longer overestimated in patients taking KTZ and that 24h-UFC*ULN are not reliable for patients taking KTZ or MTP because IA reference range values take into consideration the cross-reactivity interference not present in patients under SEI. Finally, it is noteworthy to know that the IA cross-reaction interference can come from most urinary adrenal steroid metabolites rather than only by specific precursors and that MTP- and KTZ-induced specific changes in their excretion would distinctly affect IA methods.

## Data Availability Statement

The original contributions presented in the study are included in the article/**Supplementary Material**. Further inquiries can be directed to the corresponding author.

## Ethics Statement

The studies involving human participants were reviewed and approved by Institutional Research and Ethics Committees (CEIC) from Hospital Clínic de Barcelona. The patients/participants provided their written informed consent to participate in this study.

## Author Contributions

Conceptualization: FH, GC, and AV-B. Data curation: AV-B, FH, GC, DD-C, JL-M, LB, MM, and IH. Formal analysis: AV-B, GC, FH, DD-C, JL-M, and LB. Funding acquisition: FH. Investigation: All authors participated. Supervision: FH and GC. Writing—review and editing: All authors participated. All authors contributed to the article and approved the submitted version.

## Funding

This work was supported by grants of the Carlos III Health Institute of MINECO, Spain (FIS PI 15/00859 and FIS PI19/00581). AV-B is pursuing his doctorate at the University of Barcelona with a fellowship from the Mexican National Council of Science and Technology (CONACYT) (CVU:906325).

## Conflict of Interest

The authors declare that the research was conducted in the absence of any commercial or financial relationships that could be construed as a potential conflict of interest.

## Publisher’s Note

All claims expressed in this article are solely those of the authors and do not necessarily represent those of their affiliated organizations, or those of the publisher, the editors and the reviewers. Any product that may be evaluated in this article, or claim that may be made by its manufacturer, is not guaranteed or endorsed by the publisher.
