# Supplementary figures and images for: Ketoconazole- and Metyrapone-Induced Reductions on Urinary Steroid Metabolites Alter the Urinary Free Cortisol Immunoassay Reliability in Cushing Syndrome
Source: Front Endocrinol (Lausanne). 2022 Feb 23;13:833644. doi: 10.3389/fendo.2022.833644 (PMC8905543; doi:10.3389/fendo.2022.833644)

**Supplementary Figure 1. Study design flowchart**

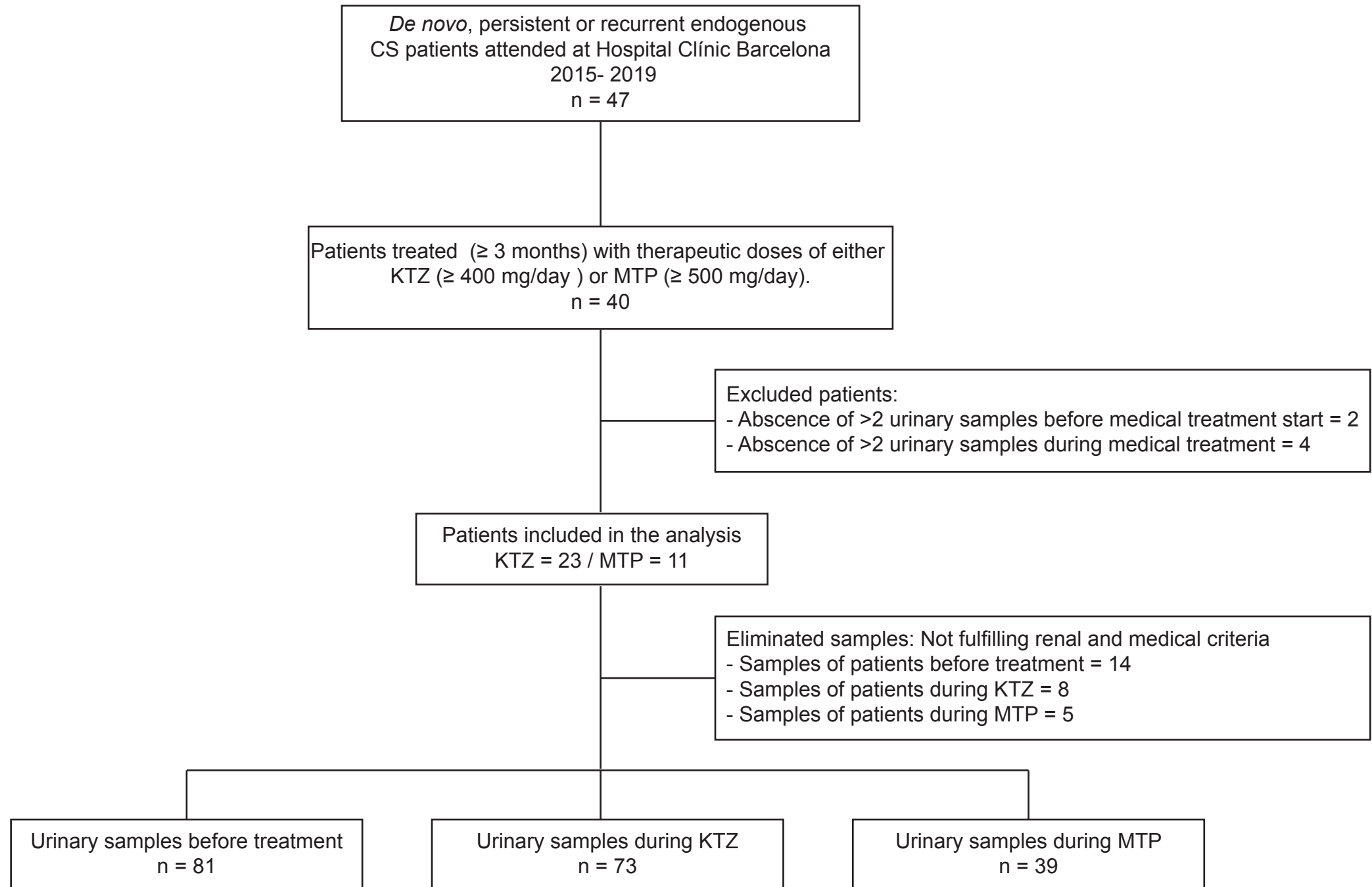

Supplement: Supplementary file 1 [file Image_1.pdf]
