# Supplementary material for: Ketoconazole- and Metyrapone-Induced Reductions on Urinary Steroid Metabolites Alter the Urinary Free Cortisol Immunoassay Reliability in Cushing Syndrome
Source: Front Endocrinol (Lausanne). 2022 Feb 23;13:833644. doi: 10.3389/fendo.2022.833644 (PMC8905543; doi:10.3389/fendo.2022.833644)
Supplement: Supplementary file 2 [file Image_2.pdf]

Supplementary Figure 2:
Ketoconazole dose association curve with the 24h-UFC ratio (IA / MS)

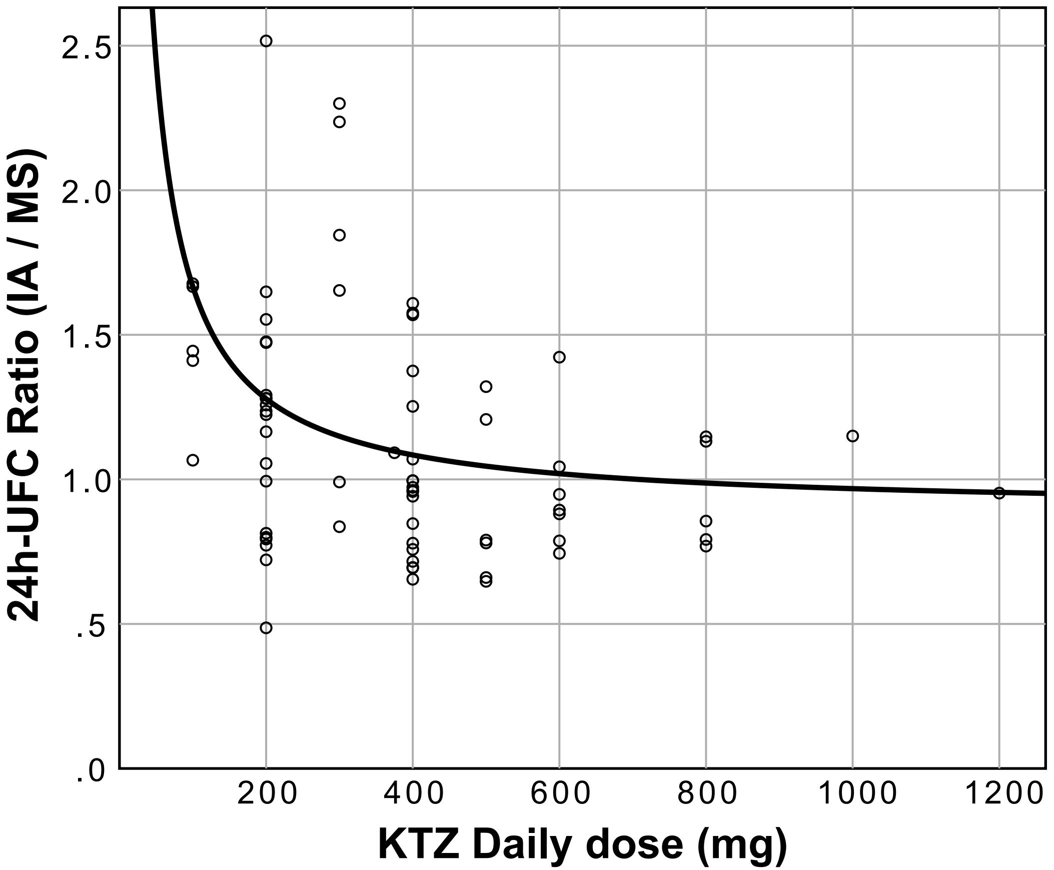

Cuadratic curve regression R2 = 0.424, p = 0.000, F = 27.28. 24h-UFC: 24 hours urinary free cortisol.
IA: Immunoassay. MS: Mass-spectometry. KTZ: Ketoconazole.
