# Supplementary material for: Ketoconazole- and Metyrapone-Induced Reductions on Urinary Steroid Metabolites Alter the Urinary Free Cortisol Immunoassay Reliability in Cushing Syndrome
Source: Front Endocrinol (Lausanne). 2022 Feb 23;13:833644. doi: 10.3389/fendo.2022.833644 (PMC8905543; doi:10.3389/fendo.2022.833644)
Supplement: Supplementary file 3 [file Image_3.pdf]

Supplementary Figure 3:
Metabolites association curves with the degree of 24h-UFC ratio

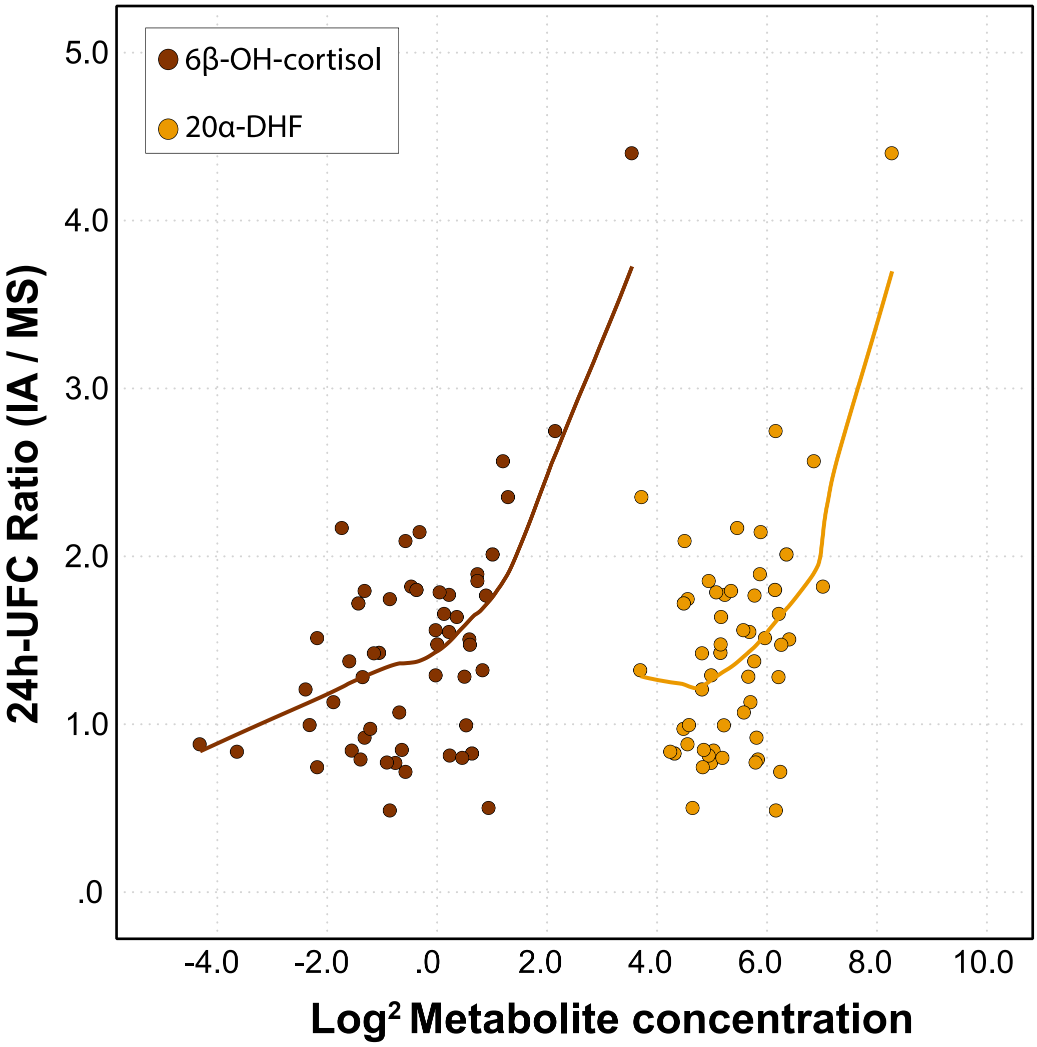

Metabolites concentrations were Log2 transforme to ensure a normal distribution.
24h-UFC: 24 hours urinary free cortisol. IA: Immunoassay. MS: Mass-spectometry.
