# Supplementary material for: Ketoconazole- and Metyrapone-Induced Reductions on Urinary Steroid Metabolites Alter the Urinary Free Cortisol Immunoassay Reliability in Cushing Syndrome
Source: Front Endocrinol (Lausanne). 2022 Feb 23;13:833644. doi: 10.3389/fendo.2022.833644 (PMC8905543; doi:10.3389/fendo.2022.833644)
Supplement: Supplementary file 4 [file Image_4.pdf]

Supplementary Figure 4:
Different metabolite changes are observed based on the dose of Ketoconazole vs Metyrapone

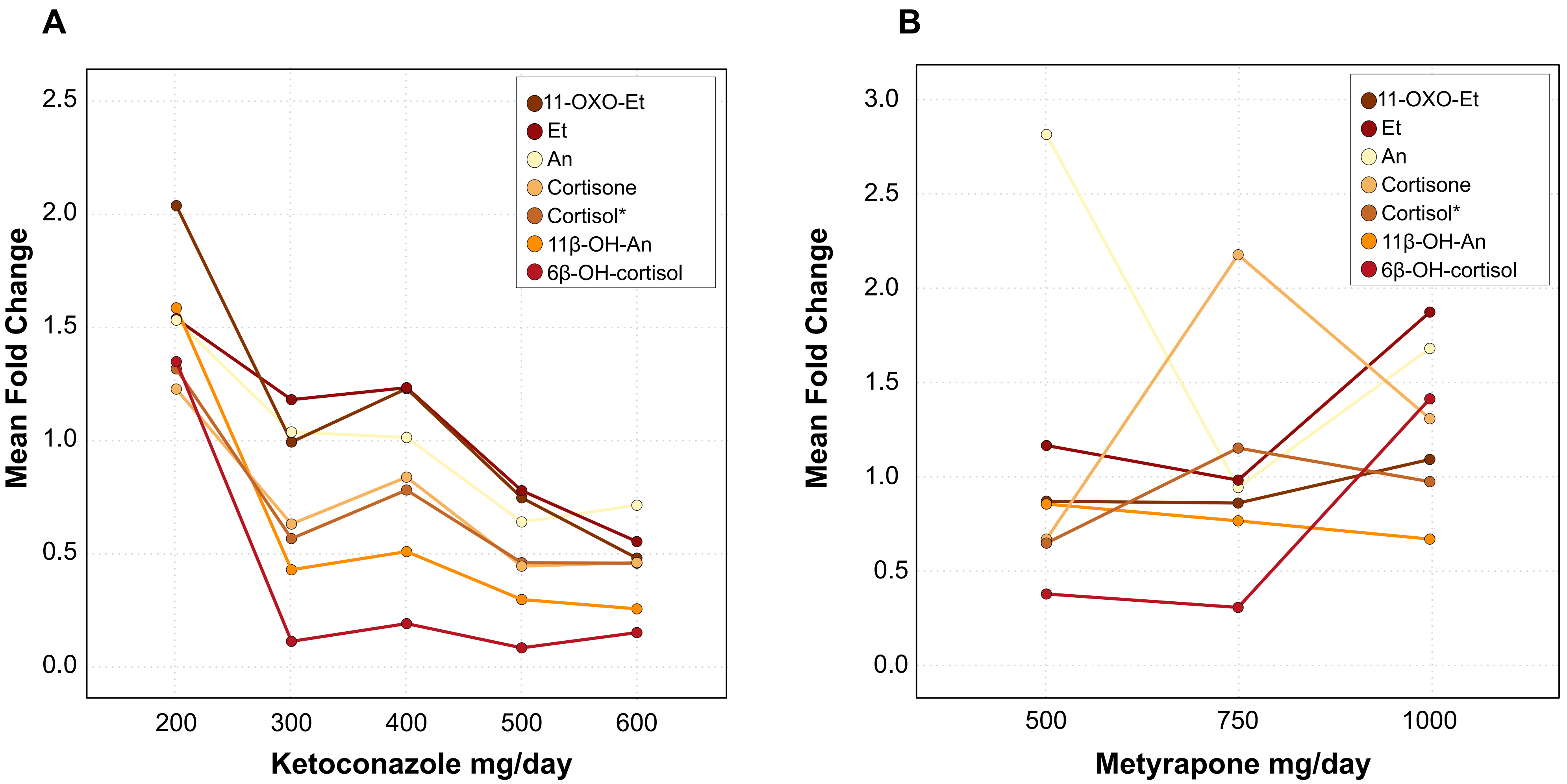

11-OXO-Et: 11-Oxo-etiocholanolone. Et: Etiocholanolone. An: Androsterone. 11β-OH-An: 11β-Hydroxy-androsterone. 6β-OH-cortisol: 6β-Hydroxy-cortisol
